# Use of Next-Generation Sequencing for Identifying Mitochondrial Disorders

**DOI:** 10.3390/cimb44030074

**Published:** 2022-02-27

**Authors:** Shafi Mahmud, Suvro Biswas, Shamima Afrose, Mohasana Akter Mita, Md. Robiul Hasan, Mst. Sharmin Sultana Shimu, Gobindo Kumar Paul, Sanghyun Chung, Md. Abu Saleh, Sultan Alshehri, Momammed M. Ghoneim, Maha Alruwaily, Bonglee Kim

**Affiliations:** 1 Department of Genetic Engineering and Biotechnology, University of Rajshahi, Rajshahi 6205, Bangladesh; suvrobiswas0@gmail.com (S.B.); shamimaafrose.ru@gmail.com (S.A.); mohasanamita26@gmail.com (M.A.M.); rhonanik7@gmail.com (M.R.H.); sharminshimu120@gmail.com (M.S.S.S.); gobindokumar38@gmail.com (G.K.P.); saleh@ru.ac.bd (M.A.S.); 2 Department of Pathology, College of Korean Medicine, Kyung Hee University, Seoul 02447, Korea; nukyung2@khu.ac.kr; 3 Department of Pharmaceutics, College of Pharmacy, King Saud University, Riyadh 11451, Saudi Arabia; salshehri1@ksu.edu.sa; 4 Department of Pharmacy Practice, College of Pharmacy, AlMaarefa University, Ad Diriyah 13713, Saudi Arabia; mghoneim@mcst.edu.sa (M.M.G.); mrowaili@mcst.edu.sa (M.A.)

**Keywords:** mitochondrial disorder, OXPHOS, mtDNA, nDNA, NGS

## Abstract

Mitochondria are major contributors to ATP synthesis, generating more than 90% of the total cellular energy production through oxidative phosphorylation (OXPHOS): metabolite oxidation, such as the β-oxidation of fatty acids, and the Krebs’s cycle. OXPHOS inadequacy due to large genetic lesions in mitochondrial as well as nuclear genes and homo- or heteroplasmic point mutations in mitochondrially encoded genes is a characteristic of heterogeneous, maternally inherited genetic disorders known as mitochondrial disorders that affect multisystemic tissues and organs with high energy requirements, resulting in various signs and symptoms. Several traditional diagnostic approaches, including magnetic resonance imaging of the brain, cardiac testing, biochemical screening, variable heteroplasmy genetic testing, identifying clinical features, and skeletal muscle biopsies, are associated with increased risks, high costs, a high degree of false-positive or false-negative results, or a lack of precision, which limits their diagnostic abilities for mitochondrial disorders. Variable heteroplasmy levels, mtDNA depletion, and the identification of pathogenic variants can be detected through genetic sequencing, including the gold standard Sanger sequencing. However, sequencing can be time consuming, and Sanger sequencing can result in the missed recognition of larger structural variations such as CNVs or copy-number variations. Although each sequencing method has its own limitations, genetic sequencing can be an alternative to traditional diagnostic methods. The ever-growing roster of possible mutations has led to the development of next-generation sequencing (NGS). The enhancement of NGS methods can offer a precise diagnosis of the mitochondrial disorder within a short period at a reasonable expense for both research and clinical applications.

## 1. Introduction to Mitochondria and Mitochondrial Disorders

Mitochondria are membrane-bound [1], intracellular and cytoplasmic organelles [2] that significantly contribute to oxidative energy metabolism in cells through the regulation of energy homeostasis by metabolizing nutrients to produce ATP which serves as a cellular energy source, and generate heat [3,4]. Mitochondria are also involved in several processes, including apoptosis, energy metabolism, steroid biosynthesis, free radical generation, reactive species generation, nucleotide metabolism, calcium homeostasis, and scavenging [4,5]. Mitochondria contribute to human metabolism through the performance of oxidative phosphorylation (OXPHOS), amino acid metabolism, iron-sulfur cluster biogenesis, fatty acid oxidation, heme biosynthesis, and the tricarboxylic acid cycle [6,7,8,9,10]. Through the OXPHOS process, mitochondria generate more than 90% of all cellular energy [11]. Mitochondrial functions rely on appropriately regulated mitochondrial assembly, dynamics, and maintenance. Mitochondria are the only organelles that encode their own genes outside of the nucleus [12,13]. The mitochondrial genome, known as mitochondrial DNA (mtDNA), encodes 37 genes among them are 13 genes involved in the OXPHOS mechanism [14]. However, nuclear genes encode most of the remaining 1500 mitochondrial proteins, which are translated in the cytoplasm and imported into the mitochondrion [12,15,16]. The coordination of both the mitochondrial and nuclear genomes is required for the appropriate mitochondrial protein expression, and numerous other metals, cofactors, and phospholipids must also be transported into the mitochondrion [6,10,17,18]. Metal ion transportation plays a crucial role in the function of mitochondria, OXPOHS, enzymatic activity, signal transduction as well as the homeostasis condition of mitochondria [19] while phospholipids are an integral part of the mitochondrial inner and outer membrane [20]. 

Mitochondrial disorders are immensely heterogeneous genetic disorders characterized by OXPHOS aberrations due to mutations in mitochondrial DNA (mtDNA) or nuclear DNA (nDNA) [21,22]. Mitochondria diseases obey the rules of mitochondrial genetics when mutations occur in mtDNA. The point mutations in mtDNA are generally maternally inherited and heteroplasmic. These can ensue with mtDNA-tRNAs, encoded proteins, or ribosomal RNA (rRNA), and therefore influence RNA processing, transcription, or replication. Nevertheless, most of the reported diseases linked with mtDNA point mutations are situated among the tRNA, ribosomal RNA (rRNA), and subunits of respiratory chain complexes (OXPHOS subunits) genes. These genes include *MT-TL1, MT-TK, MT-TA, MT-TC, MT-TD, MT-TE, MT-TM, MT-TN, MT-TS1, MT-TS2, MT-RNR1 MT-ND4, MT-ND1, MT-ND3, MT-ND2, MT-ND6, MT-ATP6*, etc. [22,23]. However, mitochondrial diseases associated with mutations in nDNA may follow Mendelian inheritance patterns [24,25,26,27]. Several nuclear genes are linked to mitochondrial disease such as; *POLG, TK2, MRPS16, PUS1, NDUFS1, NDUFS2, NDUFA1, NDUFA2, NDUFV1, NDUFV2, SDHA, SURF1, ETHE1, SCO1, SCO2*, etc. [23,28]. 

Mitochondrial diseases initially affect tissues and organs that have large energy requirements, such as the brain and skeletal muscle resulting in mitochondria-associated encephalomyopathies. Mitochondrial disorders are also often multisystemic, presenting with a variety of signs and symptoms, including heart conduction defects; cardiomyopathies; diabetes mellitus; isolated myopathies with ophthalmoplegia; central nervous system (CNS) presentations, such as stroke-like episodes: kidney or liver dysfunction: sensorineural defects, epilepsy, cognitive dysfunction, ataxia, retinal pathology, gastrointestinal dysmotility; and optic nerve atrophy [27,29].

Over 250 different mtDNA mutations were reported with the incidence of pathogenic variants in as high as 1 in 200 live births. Recent research suggest that one individual among 5000 is estimated to phenotypically express clinical manifestations of these mutations [30,31]. Another study suggested that prevalence of PMD in UK populations was estimated to be 1 in 4300 (adults and children) although this prevalence can vary according to the different ethnic community [32]. Primary mitochondrial disease (PMD) occurs due to pathogenic mutations in either nDNA or mtDNA [33]. Mutations in mtDNA often involve large-scale rearrangements and homo- or heteroplasmic point mutations. Heteroplasmic point mutations have been associated with well-established syndromes, including myoclonic epilepsy with ragged red fibers (MERRF); mitochondrial encephalomyopathy, lactic acidosis, and stroke-like episodes (MELAS); Leigh syndrome (LS); and neurogenic weakness, ataxia, and retinitis pigmentosa (NARP) [26,34]. The most common condition associated with homoplasmic mutations in mtDNA is known as Leber’s hereditary optic neuropathy (LHON). Rearrangements (single duplications or deletions) in mtDNA have been associated with the sporadic development of Kearns–Sayre syndrome (KSS), progressive external ophthalmoplegia (PEO), and Pearson’s syndrome (PS) [26,35,36,37,38,39,40]. 

The fundamental distinction between the primary mitochondrial diseases (PMDs) and secondary mitochondrial dysfunctions (SMDs) is PMD is detected when there is a pathogenic mutation in mtDNA or nDNA encoding the OXPHOS protein and SMD is detected when there is pathogenic mutation encoding a non-PMD disorder and/or scarce activity regarding an enzyme involved in a non-PMD disorder [41,42]. Moreover, PMD is generated by deletions in germline maternal mtDNA or mutation in secondary maternal and paternal nDNA mutations causing mtDNA deletions or depletion. On the other hand, SMD accompanies several pathologic processes excluding OXPHOS and including inherited diseases due to germline mutations in non-OXPHOS genes or due to secondary causes such as unfavorable environmental consequences causing oxidative stress resulting in mtDNA alterations adversely affecting mitochondria [42,43,44,45]. Additionally, mitochondrial dysfunction resulting in excess fatigue due to efficiency failure concerning the electron transport chain, reduced generation of ATP such as high-energy molecules, inadequate mitochondria number, and scarcity of necessary mitochondria substrates include Alzheimer’s disease, Parkinson’s disease, Huntington’s disease [44,46,47,48] along with other neurodegenerative, cardiovascular, and autoimmune diseases [49,50,51].

The prevalence of these mitochondrial diseases worldwide is counted as near about one in every five thousand people expressing the maximum ordinary group of inherited metabolic disorders. Contrariwise, particularly in pathogenic mtDNA mutation the range of prevalence varies between one to two hundred [52,53,54,55]. In the mtDNA patients load mutation around 70–80% in adulthood reveals neuropathy, ataxia, and retinitis pigmentosa (NARP) while 90% mutation load is expressed in infants or children exhibiting maternally inherited Leigh syndrome (MILS). Whereas nDNA mutations show Mendelian inheritance expressed in early childhood. A study indicated that between heteroplasmic and homoplasmic mutations, the heteroplasmic mutation is more frequent, with a 4.6% rate than the homoplasmic mutation, with 2.8% rates [53,54,56,57,58].

In this review paper, we will discuss the clinical characteristics of mitochondrial diseases, and how different gene or genetic variants impact the development of mitochondrial diseases. Additionally, we will discuss the currently available traditional diagnostic approaches and how the traditional approaches have failed several times while NGS technology gives an upper hand to mitochondrial diseases diagnosis [59]. Additionally, we will discuss NGS technology for diagnosing mitochondrial diseases as well as the pros and cons of these techniques with respect to diagnosis.

## 2. Clinical Features and Burden of Mitochondrial Disorders

The most usual exposure of point mutations in mitochondrial DNA (mtDNA) is “MELAS” [60]. Clinical representation comprises encephalopathy with dementia or seizures, frequent migraine headache, stroke-like occurrences exhibited before age 40, iterative vomiting, in muscle biopsy-myopathy and lactic acidosis connected with ragged-red fiber (RRF) [60,61,62]. “MERRF” is a multisystemic disease that is distinguished by symptoms such as epilepsy, weakness, myoclonus, and ataxia. A unique classical expression of this disease is the appearance of RRFs in skeletal muscle biopsies. Several other less common symptoms including peripheral neuropathy and dementia have also been exhibited [60,63,64]. Usually, young adults are affected by “NARP” and retinitis pigmentosa, sensory neuropathy, proximal weakness, etc. caused by this syndrome. “MILS” is a more acute infantile encephalopathy along with innate symmetric lesions in the brainstem and basal ganglia [37,60]. “LHON” is expressed in young adults, more often in males, which is characterized by a subacute reduction of central vision [65]. The loss of eyesight is swift and pain-free which can begin in one eye or both eyes and the colors start fading away which is called dyschromatopsia. Epidemiological research in northeastern England has presented that in 100,000 adults, the most usual mitochondrial DNA-linked disorder is LHON which has a minimum point manifestation of 3.22 [60,66,67]. Therefore, *MT-RNR1* is a gene that encodes the 12s rRNA subunit and is the mitochondrial homologue of the prokaryotic 16s rRNA. Some *MT-RNR1* variants (i.e., m.1095T > C; m.1494C > T; m.1555A > G) more closely resemble the bacterial 16s rRNA subunit and result in increased risk of aminoglycoside-induced hearing loss [68].

“KSS”, “CPEO (chronic progressive external ophthalmoplegia)” and “PS” are three dispersed conditions that are connected with single deletions in mtDNA. “KSS” is a multisystemic syndrome that is distinguished by the triad of “PEO” (abridgements of eye motions and ptosis). Frequent superfluous symptoms subsume, short stature, ataxia, endocrine malfunctions (hypoparathyroidism and diabetes mellitus) [60]. “CPEO” is a comparatively benign syndrome distinguished by proximal myopathy, “PEO”, and ptosis which is generally gradually progressive and competent with a regular life span. Exercise intolerance and dysphagia may exist [60,69,70,71]. An increasing neurodegenerative disorder that is distinguished by bilaterally symmetric lesions in the basal ganglia and/or brainstem in children and infants is known as Leigh syndrome (LS). Altered consciousness, developmental delay, seizures, pericardial effusion, failure to thrive, and dilated cardiomyopathy are exhibited in “LS” [72,73,74,75,76]. A common damning disorder of infants is “PS” which is characterized by exocrine pancreatic malfunction and sideroblastic anemia. Kidney and liver association with growth interruption and early death may occur in this syndrome [60]. Sensorineural hearing loss (SNHL) is a general medical status that can be caused by environmental or genetic factors. Approximately 80% of genetic SNHL is non-syndromic. Nonsyndromic SNHL connected with mt-DNA mutation can be exhibited as mild to severe hearing loss of both pre- and post-lingual onset. The common clinical presentation is progressive, bilateral, post-lingual hearing damage [77]. Table 1 summarizes the distinct clinical features, gene identity, variant, and associated mitochondrial diseases. 

## 3. Traditional Diagnostic Approach for Mitochondrial Disorders

Distinct clinical features, such as the age of symptom onset, which can occur from birth to adulthood, and additional features, including lactic acidosis and evidence of impaired mitochondrial respiratory chain activity, can be used to perform the initial clinical diagnosis of a mitochondrial disorder. Frequent clinical features associated with adult manifestations of mitochondrial diseases include sensorineural hearing loss, muscle weakness (dysphagia, proximal limb weakness, and dysarthria), cardiac manifestations (conduction defects, cardiac arrhythmia, and hypertrophic cardiomyopathy), endocrine abnormalities (hypoparathyroidism, hypogonadism, diabetes, and short stature), exercise intolerance, ophthalmological abnormalities (PEO, retinal pigmentary changes, optic atrophy, and ptosis), CNS involvement (focal neurological deficits, and migraine), and gastrointestinal abnormalities (constipation and pseudo-obstruction) [80].

Traditional diagnostic methods for identifying mitochondrial disorders include brain magnetic resonance imaging (MRI), biochemical screening, cardiac testing, genetic testing, clinical features, light microscopy, electron microscopy, histochemical staining, ERG, and skeletal muscle biopsy systems [84,85]. Plasma lactate levels are mildly increased in several mtDNA depletion syndromes, and similar changes in lactate levels can be detected in the cerebrospinal fluid in cases of seizures and Leigh’s disease through biochemical screening [85,86]. Echocardiogram and electrocardiogram testing can be used to detect disrupted cardiac functions associated with mitochondrial disorders. Cardiomyopathy, ventricular pre-excitation associated with mitochondrial encephalomyopathy, and atrioventricular conduction defects associated with KSS can be detected by an electrocardiogram [85,87].

**Figure 1:**
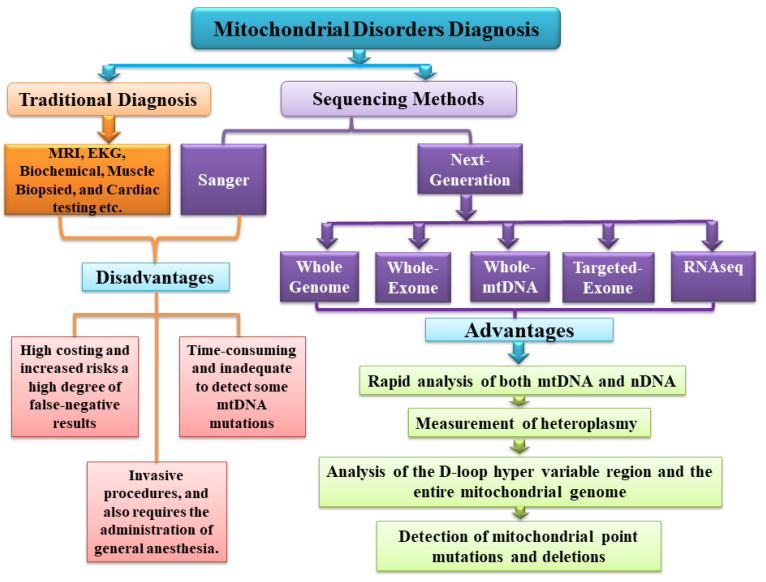
Diagrammatic representations of traditional and next-generation sequencing for identifying mitochondrial disorders.

Light microscopy and electron microscopy examinations of skeletal muscle biopsies can be used to diagnose muscle diseases associated with mitochondrial disorders based on observed histopathology. The first pathological symptom of mitochondrial disorders is the appearance of “ragged red fibers” in muscle biopsies, which can be detected using a modified Gomori trichrome stain, resulting in the rapid identification of mitochondrial disorders [85,88]. Several histochemical stains, including the detection of cytochrome oxidase (COX), nicotinamide adenine dinucleotide (NAD), and succinate dehydrogenase (SDH), can be used on frozen muscle tissues to expose mitochondrial abnormalities. Electron microscopy is useful for visualizing mitochondria for the diagnosis of structural abnormalities, whereas changes in mitochondrial aggregation can be observed at the light microscopic level, which can also be used to detect myofibrillar inclusions and nemaline rod myopathy (Figure 1).

## 4. Limitations Associated with Traditional Diagnostic Methods

Numerous limitations have been identified for traditional methods used to diagnose mitochondrial disorders. Muscle biopsies, which are necessary to observe histological and ultrastructural changes, are invasive procedures that can be damaging to high-risk or vulnerable patients, require the administration of general anesthesia, and are associated with high costs to provide an appropriate level of preparation, analysis, and exploration. In addition, appropriate sample removal, transportation, and processing are necessary for disease diagnosis, which must be performed by an expert clinician. Several patient deaths have been reported by mitochondrial experts following invasive muscle biopsy procedures.

In light microscopy, both false positive and false negative detection results are observed where this procedure exhibits normal conditions up to 50% from affected patients, and the finding is amplified over time. Age-dependent muscle biopsy for the ragged red fibers indication in young children’s and young adult’s mitochondrial disease show limited sensitivity, and limited specificity occurs in a variety of myopathies along with toxic exposures [80,85,88,89,90,91,92,93,94]. Between primary and secondary mitochondrial disease, biochemical and microscopic findings from muscle biopsy cannot be properly distinguished which leads to improper regulation and prognosis [88,89,92,95].

More often, muscle biopsy findings cannot entirely identify genetic aetiologies. Accordingly, the traditional approach to biopsy, which is Sanger sequencing based, has been able to estimate genetic diagnosis of individual genes in only approximately 11% of mitochondrial disorder patients overall [88,96,97]. In all cases, mitochondrial enzymatic abnormality detection on muscle tissue is not expressly diagnostic. Several diseases including Alzheimer’s disease, Parkinson’s disease, and other disorders such as Prader–Willi syndrome show abnormal mitochondrial enzymology which may compromise the detection of underlying preliminary pathology in a given patient [98,99].

The detection of lactate levels utilizing magnetic resonance spectroscopy is not specific to mitochondrial disorders because increased CNS lactate levels have been associated with numerous pathological processes, including hypoxia, neoplasms, and inflammation. However, the detection of lactate levels represents a non-invasive, complementary approach, along with MRS imaging, that can be used to support a mitochondrial disease diagnosis in a patient with normal-appearing MRI examinations combined with non-specific MRI findings [100]. Biochemical screening outcomes can be associated with a diverse range of suspected candidate genes [101]. Identifying mitochondrial cardiomyopathy based on cardiac specimens can be limited by non-specific morphological changes that might be associated with a vast range of storage, genetic, environmental, and metabolic causes [102].

An ever-growing archive of possible causative genes correlated with mitochondrial disease is the chief limitation of genetic testing. Thus, genetic testing has trouble rationalizing the variability regarding phenotype–genotype relationships. A further limitation of genetic testing is the requirement of accurate clinical circumstances, in conjunction with the properly chosen tissue [80,103]. Uncertainty without additional genetic tests or muscle biopsy limits the efficacy of clinical diagnosis. Moreover, costly investigations, unclear phenotype–genotype associations, the appearance of asymptomatic and oligosymptomatic mutation carriers results in lower accuracy of clinical diagnosis concerning the mitochondrial disease [80].

## 5. Role of Sequencing in Mitochondrial Disorder Detection

The entire mitochondrial genome was sequenced in 1981, and approximately 300 pathogenic mtDNA-associated mitochondrial dysfunction, point mutations, deletions, and rearrangements play a significant role in the development of multisystemic primary mitochondrial, cancer, and neurodegeneration diseases [104,105]. Therefore, a Sanger sequencing-based approach has been applied to determine the genetic contributions of individual genes; however, this approach has yielded results in only 11% of clinically prioritized patients with the mitochondrial disorder [88,96,97,106]. A total of 320 genes encoded by both the nuclear and mitochondrial genomes have been implicated in mitochondrial disorders, including 37 mtDNA-encoded, maternally inherited genes [23,88,107,108]. The determination of variant heteroplasmy levels by screening the entire mtDNA genome, and identifying mtDNA depletion through additional qPCR quantification approaches, and muscle biopsies, in tandem with testing mtDNA copy numbers, can be used to perform the genetic diagnosis of mitochondrial disorder [109]. When mutation-distinct restriction enzymes and qPCR-related approaches initially fail to detect pathogenic variants, either Sanger sequencing or chip-oriented strategies, are utilized for analyzing mtDNA [109,110,111,112]. As the mentioned methods are non-quantitative, time-consuming to identify the accurate breakpoint, and possess trouble in recognizing larger structural variation such as CNVs [113], mutation load determining next-generation sequencing (NGS) provides the ability to distinguish, characterize, and quantify point mutations along with rearrangements that require a further quantitative PCR analysis for accurate heteroplasmy level assessment regarding massive mtDNA deletions [109,114,115]. Regarding the tissue sample from individual patients needed for the genomic DNA extractions, the choice of tissue, as well as other criteria, needs to be precisely followed and modified according to the patient’s age, gender, health condition, and diseases [116,117]. Therefore, the tissue heteroplasmy needs to be observed carefully for the mitochondrial disease’s diagnosis. For example, some variants can be present in lower amounts in blood or other tissue samples making it difficult to identify any changes in biochemical abnormality and vice versa. Therefore, these unique challenges need to be addressed based on the considerations of the clinicians and the research-based functional study [118]. 

## 6. Sanger Sequencing

Sanger sequencing (SS), perceived as the gold standard and most dependable approach concerning clinical DNA sequencing over the last 20 years, narrows genetic testing through inadequate description and coverage of exons resulting in ceasing clinically relevant mutations [119]. The traditional PCR-based Sanger sequencing method can be used to analyze the whole the mitochondrial genome by utilizing varied combinations of primers [120]. Sanger sequencing can also be utilized for analyzing mtDNA deletions and point mutations in multiple genes; such as nuclear DNA originated (*TAZ, SCN5A* [121], *IDH1, IDH2, BRAF, H3F3A, TERT*) genes [122] and mitochondrial DNA originated (*MT-ND1, MT-ND4, MT-ND6* [123], *MTND5* [124]) genes, for mutations and large deletion identification [125,126]. The limitation of testing an individual or several genes at a time in conjunction with being expensive and time-consuming, in the case of varied genes testing, indicates the functional complications for the ever-increasing amounts of genetic tests regarding disease-associated genes. Though it has improved candidate gene interrogation and the number of recognized disease genes through specific genes sequencing, it is less efficient due to overlapping or alike clinical presentations [119,127,128].

## 7. Significance of NGS for the Detection of Mitochondrial Disorders

The NGS method is a rapid identification method for the detection of mitochondrial mutational disorders which can be caused by one of several genes and allows hundreds of genes in parallel and the detection of mutations [129]. The NGS identified 15–20 new genes in every year and more than 1500 nuclear genes are associated with mitochondrial function [109]. The mutations are associated with mitochondrial disorders and allow for the recognition of genotype–phenotype relationships [23,88,108,130]. The NGS approach has revolutionized the molecular-level understanding of complex mitochondrial disorders, resulting in improved sequencing outcomes for both the whole genome and for targeted coding sequences, at lower cost and with improved accessibility, allowing research labs to more readily perform large-scale sequencing studies [131,132]. Several approaches of NGS technologies, including WGS (whole-genome sequencing), WES (whole-exome sequencing), whole mtDNA sequencing, mitoexome (targeted-exome sequencing), and RNAseq, have favorably served in diminishing the apprehension around variants and disease, whose advantage is conditioned on the expense per raw base, precision by the raw base, throughput by apparatus, and read length through the independent read [131,133,134,135].

## 8. Whole-Genome Sequencing

WGS (whole-genome sequencing) refers to the complete genome sequencing working flow to reveal the sequences of an organism’s genome at the same time [136]. Approximately 50% of disease-causing variants along with 30% of variants that influence gene expression positioned in deep intronic regions according to transcriptomic analysis form lead to the need to inspect non-coding-region-linked variant pathogenicity via an approach such as WGS [137,138,139,140,141]. In WGS, the genome is discerned in a tiered technique after determining an index containing a few hundred to few thousand genes that are appropriate according to the patient’s phenotype utilizing databases, including “Kyoto Encyclopedia of Genes and Genomes” [https://www.genome.jp/kegg/ accessed on 1 February 2022 in conjunction with “Online Mendelian Inheritance in Man” [https://www.omim.org/ accessed on 1 February 2022; [142]. Whole-genome sequencing (WGS) can identify variants within the entire genome, providing distinct diagnostic advantages, including the ability to detect low-level heteroplasmic disease variants and intragenic nuclear DNA deletions, and can be applied to attain comprehensive mtDNA sequencing [143]. It is a notable advantage of whole-genome sequencing that it can identify the changes emerging in either intron or regulatory regions, which remain uncaptured during whole-exome sequencing [131]. 

Three novel mitochondrial disorder-causing genes, *COQ5*, *TIMMDC1*, and *COX6A1,* are characterized by the detection of a 30-base-pair untranslated region (UTR) duplication, a profound intronic variant, and an intronic deletion, respectively, which can be identified using WGS [137,144,145,146]. Although few descriptions of WGS have been reported for the large-scale characterization of mitochondrial disease cohorts, diagnostic WGS provides the possibility of sequencing a genome with a high depth of coverage, for example, 40–60 fold, allowing for the identification of nuclear genetic variants [134,147]. The average detection of variant mtDNA heteroplasmy from a single WGS analysis of blood samples that examines both the mitochondrial and nuclear genomes can ensure a slightly improved detection (40%) of mitochondrial disorders compared with whole-exome sequencing (WES, 38%) [134,147,148,149]. Often, WGS is preferred because it reanalyzes negative cases as it captures data of every gene without considering disease-causing status, proven through the 10% to 20% diagnostic progress of 1000 reanalyzed specimens predominantly to find novel disease-associated genes [137,150,151].

## 9. Whole-Exome Sequencing

The whole-exome sequencing or WES refers to sequencing for the protein-coding regions in a genome [152], attractive for clinical applications due to affordability in variant determinations, and pathogenic mutation identifications [153,154]. WES is an impartial and comprehensive strategy for mtDNA analysis, able to provide more than a 35–68% diagnosis of genetic disease along with whole-mitochondrial-genome sequencing (WMGS), which is able to recognize novel disease genes and mutations [88,108,155]. WES examines the protein-coding sequence of the genome that covers almost 400 protein-altering unique variants to spread genotypic heterogeneity, confirming the comprehensive phenotypic heterogeneity of genes of recognized disorders [38,137]. The analysis of mtDNA by WES data does not require the sequencing of the entire mtDNA genome. WES can instead be used to capture mtDNA sequences in off-target reads, with a high accuracy (>99%) and recall (>95%), and the ability to differentiate heteroplasmy (>10%), which is advantageous among pediatric populations with higher recurrent nDNA mutations [137,156]. Though commercially available whole-exome capture kits lackbaits targeting the mitochondrial genome, it provides complete coverage concerning mtDNA which is stated as off-target capture [131]. Moreover, the limitations of WES being time consuming and complex data interpretation can be resolved by filtrating genetic variants via the application of virtual gene panels [157]. The WES approach allows data re-investigation by utilizing enhanced data handling and annotation or assessing novel genes correlated with MD, and hence it is favorably executed as a cost-effective molecular diagnostic approach for any genetic disorders such as mitochondrial disorders [136,157]. The off-target WES approaches are utilized predominantly in the diagnosis of MD in children offering >90% positive outcomes, provide >30-fold mean read coverage along with a marginally lower error rate in comparison to Sanger sequencing determining the mtDNA sequence in tandem with maternal ancestry and quality control estimation, ethnic origin identification and genetic disease association [158,159]. Though there are no baits targeting mtDNA in business whole-exome capture kits, the mitochondrial genome can be scrutinized by whole-exome capture kits, resulting in complete mtDNA coverage as plenty of mtDNA copy is available [131,160]. However, a mean level of heteroplasmy implementing a negative result during WES does not take into account the absence of mt-DNA variants in patients, and yet a recognized variant by WES may not dwell amid the coding regions of the mitochondrial genome [137]. Last but not the least, an infant having a recessive disorder for the mutations concerning *TWNK* resulting in mitochondrial DNA depletion diagnosed by WES is not a fit for liver transplantation despite acute liver failure, which indicates that WES aids in the critical important treatment decision [142].

## 10. Whole-mtDNA Sequencing

Mitochondrial DNA is regarded as being more prone to alternations compared to nuclear DNA [161,162], and these abnormalities in the mitochondrial genome lead to the mitochondrial dysfunctions and multiple diseases [163]. Whole-mtDNA sequencing is a reliable NGS sequencing strategy for the high-quality sequencing of mtDNA, allowing for the possibility of identifying mitochondrial disorder-associated variants [164]. Whole-mtDNA sequencing can be used to screen the entire mtDNA sequence, allowing for the estimation of heteroplasmy levels using easily accessible DNA specimens obtained from blood and urine samples. However, mtDNA heteroplasmy, copy numbers, and replication are not uniform across tissues due to differences in regional energy requirements, and negative whole-mtDNA sequencing results from urine or blood samples must be verified by tissue samples, including skeletal muscle or urinary epithelial cells [137,165]. Therefore, the whole-mtDNA sequencing workflow alone is not sufficient for the mitochondrial diseases that result from pathogenic mutations where combinatorial sequencing approaches from other tools requires for the establishment of molecular diagnostics [131]. Through whole-mtDNA sequencing method, whole mitochondrial DNA can be sequenced and any variant can be located and give a proper appraisement in terms of heteroplasmy levels. In several mitochondrial diagnostic centers, at first mtDNA is sequenced to deduct mitochondrial variants [134].

## 11. Targeted-Exome Sequencing

Molecular diagnoses based on approximately 100 genetic loci and novel disease genes associated with canonical mitochondrial disorders can be identified using targeted-exome sequencing [166,167,168]. The utility concerning targeted-exome sequencing was notable when a study of 102 patients with presumed mitochondrial disorders was published, and during this period, 100 genetic loci were identified. The fact is the number of identified genes regarding mitochondrial disease is increasing, which is approximately 320 at the time this review article was written [166]. However, large-scale identifications of variants or rearrangements in this technique are less useful; pre-defined gene or common translocating regions are more useful. The copy number of changes detections is much more precise in whole-genome or whole-exome sequencing compared to targeted-exome sequencing [169]. Therefore, a part of the genome sequence may not be captured during WES, where changes occur in the intron region or regulatory region that may be left undetected by WES. However, the customizations of the target capture, and reduced size of the target improved the sequence coverage [131]. The generation of short-sequence fragments using the currently available methods can make the identification of insertion–deletion mutations difficult and resequencing by increasing the number of sequence reads has been shown to improve targeted-exome coverage. Therefore, targeted-exome sequencing represents a time- and cost-efficient sequential analysis for mtDNA, and like WES and WGS this technique is able to differentiate phenotypic overlaps between mitochondrial disorders and distinct genetic syndromes, recognize de novo variants, filter candidate variants, and facilitate haplotype phasing [166]. Targeted-exome sequencing, which is also known as “mitoexome”, is accepted mainly for the diagnosis of Mendelian disorders and conventional cytogenic and it exhibits very strong coverage for the chosen gene panel. This process alleviates variants of unfamiliar significance and the number of ancillary discoveries. The targeted-exome sequencing method aids in both inspecting genes that potentially cause mitochondrial disorders and inquiring genes that are thought to perhaps cause disease [134,170]. This method is a time-effective and cost-efficient substitute to sequential, the traditional testing of the particular nuclear genes and mtDNA. A suitable molecular diagnosis can be founded shortly in a deficiently invasive system through this method [166]. This is a favored process for diagnostic usage, and experiment-based validation will be needed to exhibit novel disease genes pathogenicity [131].

## 12. RNA Sequencing

The quantity and the presence of RNA in biological samples are revealed through RNA-seq or RNA sequencing technology [171]. Although RNA sequencing (RNAseq) is a potential alternative, no validation has been presented regarding the identification of mitochondrial variants using RNAseq analysis. However, RNAseq can precisely detect mitochondrial single-nucleotide polymorphisms, with higher medial profundity for mitochondrial regions from RNAs captured from the mitochondria during RNA library development. Moreover, RNAseq provides great coverage of coding regions and aligns more extended reads for the mitochondrial genome [172]. In addition, this RNA sequencing approach drastically enhances detection sensitivity along or in parallel with DNA sequencing, providing information on an entire expressed genome sequence with multiple outstanding functions such as detecting mono-allelic expression, divergent gene expression, sequence variant in non-coding regions, and aberrant splicing to define an emerging new disease. A statistical analysis found that patients suspected of having a mitochondrial disorder who underwent RNA sequencing of a fibroblast cell line exhibited diagnostic outputs of about ten percent, wherein muscle samples delivered higher outputs (35%), presenting both aberrant splicing and splice defect range detection [147,173,174].

## 13. Case Examples

The statement that the next-generation sequencing or NGS provide both more precise and effective diagnosis of mitochondrial disorder than the traditional approaches can be strengthened by a few previous patients’ diagnosis case examples. 

In mtDNA patients, a load mutation of around 70–80% in adulthood reveals Neuropathy, Ataxia, and Retinitis Pigmentosa (NARP), while 90% mutation load is expressed in infants or children exhibiting Maternally Inherited Leigh syndrome (MILS) [175]. An investigation included four patients carrying three distinctive mitochondrial disorders, including Leigh syndrome rendered by an *MT-ATP6* gene mutation and mitochondrial complex I deficiency rendered by an *NDUFV1* gene mutation, in conjunction with coenzyme Q10 deficiency rendered by mutations regarding the *COQ* gene. Traditional diagnostic testing strategies were incompetent to specify a molecular-based diagnosis. In all the patients, exome sequencing assessing both the nuclear genome in tandem with the mitochondrial genome was conducted in a prompt and cost-effective manner [176]. 

Another analysis conducted in Estonia for patients with a suspected but unsolved mitochondrial disorder (MD) found the efficacy of NGS, precisely, whole-exome sequencing (WES), in clinical practice. Among the 28 patients, a disease-causing variant was uncovered in 16 patients, whereas a disease-causing variant in multiple genes regarding MD in four patients was located, confirming a satisfactory value of WES having the diagnostic yield as 57% [177]. 

In addition, in another investigation, the traditional diagnostic approach revealed a diagnosis concerning mitochondrial disease in 29.50% or 115 of patients among 390 patients. Then, 116 patients (36 patients retaining mitochondrial diagnosis, in tandem with 80 patients retaining no diagnosis) underwent nuclear whole-exome sequencing or nWES. The nWES revealed that one patient amid 36 traditionally predetermined MITO patients harbored no known pathogenic mtDNA variant, whereas one patient amid 80 traditionally predetermined NO-Dx patients retained a mitochondrial diagnosis. This study demonstrated that NGS approaches such as nWES provide a more accurate diagnosis regarding MD than the traditional approaches [178]. 

## 14. Challenges and Overcome of NGS Data Analysis Tools

There are many challenges associated with next-generation sequencing data analysis tools, such as the difficulty in processing a huge volume of data, multiple steps for data analysis [179], and several sequential operations [179]. The key challenge in the NGS data analysis pipeline is the sheer volume of raw data of every sample; sometimes, the whole-genome raw data can be up to 250 GB, where high-computational accelerating resources are required for standard data analysis pipelines [180]. Additionally, finding appropriate computational tools and algorithms for NGS analysis pipelines, especially for newly designed pipelines, is difficult and time consuming [181]. The selection of particular tools and also the result of the newly developed tools need to be quantified as parallel to existing or alternative workflows [182,183]. The magnitude of sequence data acceleration is required to speed up the entire NGS workflow where optimized parallelization is used [184]. The input data are split into small and equal portions followed by an alignment parallel to array jobs [184] or using multithread for the distribution of data, or across the multiple compute mode using message passaging interface [185]. The data security for large genomic datasets and maintaining the confidentiality is one of the key aspect where storing metadata with permission-based access and individual data barcodes may help to protect the data [180].

## 15. Mitochondrial DNA and Mitochondrial Disease Databases

Several databases including HmtDB (https://www.hmtdb.uniba.it/ accessed on 1 February 2022), MITOMAP (https://www.mitomap.org/foswiki/bin/view/MITOMAP/WebHome accessed on 1 February 2022, and MSeqDR (https://mseqdr.org/ accessed on 1 February 2022. are currently available, which aids in the annotation of the mtDNA results derived from the NGS techniques.

Human mitochondrial genome sequences from healthy and disease phenotypes are included in the HmtDB database. The database is used by clinicians as well as population geneticists for undertaking the task of evaluating the pathogenicity of definite mtDNA mutations. Guided by the advent of next-generation sequencing (NGS), the database currently offers thousands of genomes sequence records which are obtained from both pathologic and healthy samples and it provides a straight connection to high-performance pipelines following the analysis of human mtDNA, which constantly needs variant annotations with a human mtDNA reference. Nowadays, in HmtDB database 32,922 mitochondrial genomes are publicly attainable, along with 1427 mitochondrial genomes from exome data [160,186], which is propagated by the 1000 Genomes Project [187,188]. Samples determined to be “healthy” attained from population studies and “pathologic” genomes were obtained from individuals invaded by mitochondrial diseases or some other narrated clinical circumstances. HmtDB is currently considered as the most liable resource of exhortation for mtDNA variants, assessing the originality, polymorphic characters or even the deduced pathogenicity of mtDNA variants [189].

Another magnificent database entitled MITOMAP exhibits human mtDNA variation and the gene sequence of mitochondrial variation, which make the database the most convenient web-based search tool to explore human disease and evolution. The MITOMAP database tools provide information of possible mutation that is linked to disease mutation such as rRNA or tRNA mutation, coding and non-coding or control region mutations which are methodized by both mtDNA location and phenotype. Three classified variant sequences, such as adaptative, neutral, and pathogenic sequences, are produced which raised the drastic mutation rate of human mtDNA [190]. The pathogenic mutation containing table and a nuclear-mtDNA pseudogene (NUMT) database are elaborately expanded in coding region sequences based phylogenetic tree through the MITOMAP database which shows the mutation identified with others exhibiting several clinical statements. NUMT pseudogene variant sequences are erroneously thought to cause disease through inadvertent sequencing, which is easily restrained by scanning human NUMT data through the MITOMAP database [190,191,192]. 

MSeqDR, the Mitochondrial Disease Sequence Data Resource, is an encyclopedic and centralized phenome and genome bioinformatics web portal constructed by the Mitochondrial Disease Sequence Data Resource Consortium that securely assembles and conveys data regarding rare diseases and causative mutations combining community learning through a global measure curated by 100+ mitochondrial disease experts by scrutinizing data (single gene, variant, region, diseases, phenotype, variants or genes, WES or WGS, and clinical data and raw sequence in FASTQ, FASTA, and BAM formats) of mitochondrial and nuclear DNA, with the help of freely accessible online bioinformatics-based mtDNA tool suite [193]. The web portal is developed for mitochondrial disses in tandem with mtDNA mutations to ameliorate research investigations and clinical diagnosis concerning particular patient genes, genomes, variants, and phenotypes, constructing a vast metapopulation reference regarding allele frequencies of mtDNA variant that offers position-specific variant data for about 93,000 people located at some positions [193,194]. In the case of genotype–disease association, the MSeqDR-LSDB seems to be a constantly curated database concerning 280 mitochondrial diseases, including over 15,000 pathogenicity-assessed variants derived from 1500 genes associated with mitochondrial roles, incorporating about 290 comprehended disease genes [195]. Furthermore, generic annotations are derived from VEP (Variant effect predictor) and dbNSFP, whereas the hierarchical tree-form disease investigation with the assistance of expert-curated data from HmtDB and MITOMAP, which offer allele frequencies measuring over 47,000 germline mitogenomes, in conjunction with pathogenicity and disease categories from MSeqDR, HmtDB, Mitomap, and ClinVar, is carried by mvTool, known as a prevalent one-stop mtDNA-related variant analysis and annotation web aid, dependent on the MSeqDR [196,197]. Hence, the mvTool is utilized for deep annotation and pathogenicity analysis; likewise, Phy-Mer for haplogroup determination, MToolBox for heteroplasmy calculation, and haplogroup determination are available as well, along with HmtDB, MITO Master, and Mito TIP Trna Scoring as mtDNA analyzing bioinformatic tools [195]. Therefore, the data from the NGS approaches can be utilized for annotation by using as an input in the database named MSeqDR.

## 16. Future of the NGS in Mitochondrial Disorders

Next-generation sequencing is being modernized continuously through the progressing research and reforms for endeavoring more economical, more agile, and more precise sequencing approaches to revolutionize the genomics arena of research and clinics, providing a wider utilization spectrum [198]. The application of Sanger sequencing to the diagnosis of multigenic disorders is cost prohibitive, technically difficult, and time consuming, whereas NGS represents a cost-effective method that can provide 100 times as much data as Sanger sequencing, including targeted gene sequencing for multigenic disorders, with a high accuracy and efficiency. The inability of Sanger sequencing to reliably identify low levels of mtDNA heteroplasmy has resulted in the increased applications of high-throughput NGS during prenatal genetic screening and genetic counseling due to its ability to identify low levels of mtDNA heteroplasmy [134,199]. Despite having its own limitations, it functions as a gold standard for the validation of NGS data to differentiate between heteroplasmic variations as well as poor-quality reads in the data [200]. 

However, genome sequencing is not a first-line strategy for mitochondrial disease diagnosis because data interpretation can be difficult and expensive. Cost reductions and improved methodological approaches can lead to genome sequencing strategies becoming more plausible approaches for distinguishing mitochondrial diseases [143]. KSS combined with PEO is generally undetectable in blood samples but can be identified in post-mitotic skeletal muscle, requiring the performance of tissue biopsies if genetic testing is inconclusive [137,201]. Currently available commercial kits inadequately capture disease-related regions, requiring the development of customized target capture methods, and targeted region size reductions are recommended to provide better sequence coverage through targeted-exome sequencing. However, RNAseq does not provide coverage of mtDNA in non-coding regions, in addition to featuring a high false-positive rate for mitochondrial variant detection, which must be resolved to provide a more precise recognition of mitochondrial single-nucleotide polymorphisms [172].

The use of NGS has resulted in exponential improvements in the determination of genetic explanations for rare diseases and heterogeneous disorders due to the ability to recognize de novo mutations by examining complete or favored regions of the genome. As NGS becomes increasingly implemented for both research and clinical applications, the rapid diagnosis of disease following whole-genome investigations can be achieved in less time and with moderate expense compared to current diagnostic approaches. However, coverage and precision of less than 100% of termination variants have generated concern regarding variant interpretation, as more than one individual candidate variant is often detected. Moreover, the proportions of false-positive results, the determination of diagnostic criteria, consistent quality control, variant analysis, the supervision of unaffected probands, and ethical objections must be overcome prior to the large-scale implementation of NGS in the diagnosis of mitochondrial diseases [202]. The targeted-genome sequencing largely enables the detection of genetic-based diagnoses of mitochondrial diseases, but WES or WGS approaches generate more comprehensive data to analyze the mtDNA, new variants, novel disease-causing genes, and monogenic phenocopies, and the is are rapidly decreasing for these sequencing technologies [88]. 

## 17. Conclusions

Diverse mitochondrial disorders are associated with clinical features that can be difficult to differentiate and diagnose due to an ever-increasing number of suspected genes and complicated data analyses. Many traditional diagnostic approaches, such as MRI, cardiac testing, genetic testing, and biochemical screening, are currently used to diagnose these disorders, but each of these approaches is associated with disadvantages. To diagnose the mitochondrial diseases patients, no single laboratory or diagnostic test are gold standard for the confirmation the diagnosis of a mitochondrial disease. NGS represents an improvement over older diagnostic approaches, with fewer diagnostic difficulties along with the pathological testing. The NGS approach can favorably identify several novel genes that have been identified in association with mitochondrial disorders in the past decade, providing a time-efficient, cost-effective, and precise detection of mutations on a large scale. Therefore, NGS has broadened the genomics arena for the detection of mitochondrial disorders, and numerous enhancements are possible by applying various strategies compared with other sequencing and older diagnostic approaches.

## Figures and Tables

**Table 1:** Summary of clinical phenotypes, genetic mutations, and common clinical features of mitochondrial disorders.

Clinical Phenotype	Genetic Mutation	Most Common Clinical Features
MELAS Syndrome [60]	Mostly linked with the mutation designated m.3243A > G in the *MTTL* gene. Another notable point mutation is m.13513G > A in (*MTND5*) gene	Migraine, hearing loss, exercise intolerance, growth failure, diabetes, gastrointestinal disturbances, cardiopathy, and ophthalmoparesis
MERRF Syndrome [60]	Typical m.8344A > G mutation in the gene denominated as *MTTK*	Fatigue, ataxia, myoclonus, seizure, weakness in the muscle, ptosis, numerous lipomas, and hearing damage
NARP Syndrome [60]	Caused by 70% mutation in the gene entitled *MT-ATP6*	Seizures, dementia, ataxia
MILS Syndrome [60]	Caused by 90% mutation in the gene recognized as *MT-ATP6*	Basal ganglia and brainstem lesion
LHON Syndrome [60]	Mutations in three genes m.3460G > A in the gene identified as *MTND1*, m.14484T > C in the gene acknowledged as *MTND6* and m.11778G > A in *MTND4* of complex I (ND genes)	Dyschromatopsia, pseudoedema
Kearns Sayre syndrome (KSS) [78]	Single large-scale deletion	Pigmentary retinopathy, progressive external ophthalmoplegia with ptosis, and cardiac conduction flaws
Progressive external ophthalmoplegia (PEO)/(CPEO) [78,79]	Single large-scale deletion; mutations known as m.3243A > G, m.3243A > T, m.4298G > A, m.4308G > A, m.5690A > G, m.5703G > A, m.12276G > A, m.12294G > A, m.12315G > A, m.12316G > A involving *MT-TL1, MT-TI*, *MT-TN*, *MT-TL2* genes	Impaired eye movements, ptosis
Leigh’s disease [80]	Point mutation occurs in the subunit of protein	Lesions in basal ganglia, psychomotor interruption, problems in movement, lactic acidosis
Pearson syndrome [78]	Single large-scale deletion	Sideroblastic anemia, short stature, exocrine pancreatic insufficiency, and failure to thrive
Epilepsy [81,82]	Recessive *POLG* mutations; mutations recognized as m.8344A > G in *MT-TK*, m.3243A > G in *MT-TL1*, m.611G > A, and m.583G > A in *MT-TF* gene	Refractory status epilepticus, migraine, psychiatric
Nonsyndromic sensorineural hearing loss [83]	Point mutations at m.1555A > G, m.7445A > G, m.1494C > T, and m.7511T > C in *MT-RNR1*, *MT-TS1* genes	Moderate improving hearing loss, acute deafness

## Data Availability

Not applicable.
